# Biotechnological response curve of the cyanobacterium *Spirulina subsalsa* to light energy gradient

**DOI:** 10.1186/s13068-023-02277-4

**Published:** 2023-02-19

**Authors:** Luigi Pistelli, Angelo Del Mondo, Arianna Smerilli, Federico Corato, Clementina Sansone, Christophe Brunet

**Affiliations:** 1 Stazione zoologica Anton Dohrn, sede Molosiglio Marina Acton, via ammiraglio F. Acton, 55., 80133 Naples, Italy; 2 Stazione zoologica Anton Dohrn, villa comunale, 80121 Naples, Italy

**Keywords:** Vitamins, Carotenoids, Polyphenols, Spectral light, Microalgae, Biomass, Light energy

## Abstract

**Background:**

Microalgae represent a suitable and eco-sustainable resource for human needs thanks to their fast growth ability, together with the great diversity in species and intracellular secondary bioactive metabolites. These high-added-value compounds are of great interest for human health or animal feed. The intracellular content of these valuable compound families is tightly associated with the microalgal biological state and responds to environmental cues, e.g., light. Our study develops a *Biotechnological response curve* strategy exploring the bioactive metabolites synthesis in the marine cyanobacterium *Spirulina subsalsa* over a light energy gradient. The Relative Light energy index generated in our study integrates the red, green and blue photon flux density with their relative photon energy. The *Biotechnological response curve* combined biochemical analysis of the macromolecular composition (total protein, lipid, and carbohydrate content), total sterols, polyphenols and flavonoids, carotenoids, phenolic compounds, vitamins (A, B_1_, B_2_, B_6_, B_9_, B_12_, C, D_2_, D_3_, E, H, and K_1_), phycobiliproteins, together with the antioxidant activity of the biomass as well as the growth ability and photosynthesis.

**Results:**

Results demonstrated that light energy significantly modulate the biochemical status of the microalga *Spirulina subsalsa* revealing the relevance of the light energy index to explain the light-induced biological variability. The sharp decrease of the photosynthetic rate at high light energy was accompanied with an increase of the antioxidant network response, such as carotenoids, total polyphenols, and the antioxidant capacity. Conversely, low light energy favorized the intracellular content of lipids and vitamins (B_2_, B_6_, B_9_, D_3_, K_1_, A, C, H, and B_12_) compared to high light energy.

**Conclusions:**

Results of the *Biotechnological response curves* were discussed in their functional and physiological relevance as well as for the essence of their potential biotechnological applications. This study emphasized the light energy as a relevant tool to explain the biological responses of microalgae towards light climate variability, and, therefore, to design metabolic manipulation of microalgae.

**Supplementary Information:**

The online version contains supplementary material available at 10.1186/s13068-023-02277-4.

## Introduction

Microalgae, a group rich of 30,000 known species both prokaryotes and eukaryotes [1] are one of the solutions to the challenges and requirements of the modern bioeconomy [2–4]. The interest in microalgae in the context of eco-sustainable development is growing, deserving potential roles in human health, feed, aquaculture, biomaterials, energy, and environmental protection. Both the human health and feed sector could profit from the remarkable microalgal secondary metabolites content and diversity [3, 5], such as PUFAs, vitamins, carotenoids or polyphenols [6–8]. Cells regulate secondary metabolite synthesis is regulated by cells in response to driving forces inducing protection, signaling, defense or repair mechanisms [9, 10]. The cellular level of secondary metabolites depends thus on both cell and environment constraints, i.e., on the organismal fitness within its environment and its variations. It is noteworthy that light is one of the main ecological axes for photosynthetic organisms in the marine environment [11, 12]. Microalgal cells inhabiting the pelagic system and, therefore, moving up and down along water column, experience changes in photon flux density (PFD) and spectral variations [12]. These parameters are interconnected: both spectral composition changes and PFD variations determine a change in total light energy, according to the different energy levels of the photons characterizing the different wavelengths (red, green, or blue). Microalgae are, therefore, adapted to cope with intricate changes through efficient signaling involving blue and red photoreceptors [13–17].

This adaptive trait lays the foundation for considering light as a *metabolic designer* in controlled microalgal productive plants to manipulate secondary metabolite synthesis. Our study develops a *Biotechnologically Response Curves* (B.R.C.) analysis, i.e., the quantification of bioactive secondary metabolites in microalga biomass grown over a light energy gradient. Our study integrates the spectrally dependent photon energy, which is generally not considered in physiological/biological studies into the characterization of the different light climates, proposing the so-called Relative Light energy index (Rel_LEi, in µmol m^−2^ s^−1^), defined as follows:

$$ {\text{Rel}}\_{\text{LEi}} = \sum {({\text{PFD}}_{\lambda } \cdot {\text{Rel}}\_{\text{eV}}_{\lambda } )} $$

where PFD_*λ*_ was the photon flux density of the red, blue or green wavelength under the different light climates and Rel_eV_*λ*_ was the relative energy in electron volt of the red, blue or green photon.

The relative light energy index (rel_LEi) is a proxy that combines in one parameter the total PAR photon flux density, the photon flux density of the three wavelengths together with the photon energy of each wavelength. Higher the wavelength of a photon, lower its energy (red: 1.8 eV; green: 2.3 eV; blue: 3.1 eV). Although red has low energy, it is enough to reduce NADPH from water, one of the light-dependent reactions belonging to the “Z scheme”. However, the different photonic energies of red or blue spectra modulate monophotonic excitation or photoredox capacity [18], that might, therefore, modify heat dissipation from chlorophyll *a* and/or potential photodamage. The Biotechnologically Response Curve enclosed the biochemical characterization of the biomass regarding the macromolecular composition (total protein, lipid, and carbohydrate content), total sterols, polyphenols and flavonoids, carotenoids, phenolic compounds, vitamins (A, B_1_, B_2_, B_6_, B_9_, B_12_, C, D_2_, D_3_, E, H, and K_1_), phycobiliproteins, and the antioxidant activity of the extracts. Growth ability and photosynthesis were also measured under the different light climates.

The microalgal model targeted by our study was the marine cyanobacterium *Spirulina subsalsa* on which some biological data were already available [19–24]. In a microalgal biotechnological prospecting context it is urgently necessary to expand the low number of used species compared to the huge biodiversity, for instance for health benefit outcomes [25, 26]. In addition, due to the on-going and prospected global water crisis, microalga species requiring marine instead of freshwater water to grow might represent the best target for large-scale industrial production.

This study focussed on the human health-derived interests of the marine *Spirulina subsalsa* with the aim to illustrate its bioactive secondary metabolites profile over a light climate gradient. The latter was defined thanks to the description of a relative Light energy index, also exploring the role of spectral-dependent photon energy on the nutritional and antioxidant status of *S. subsala*.

## Results

### Photosynthetic properties, biomass yield and composition

**Fig. 1:**
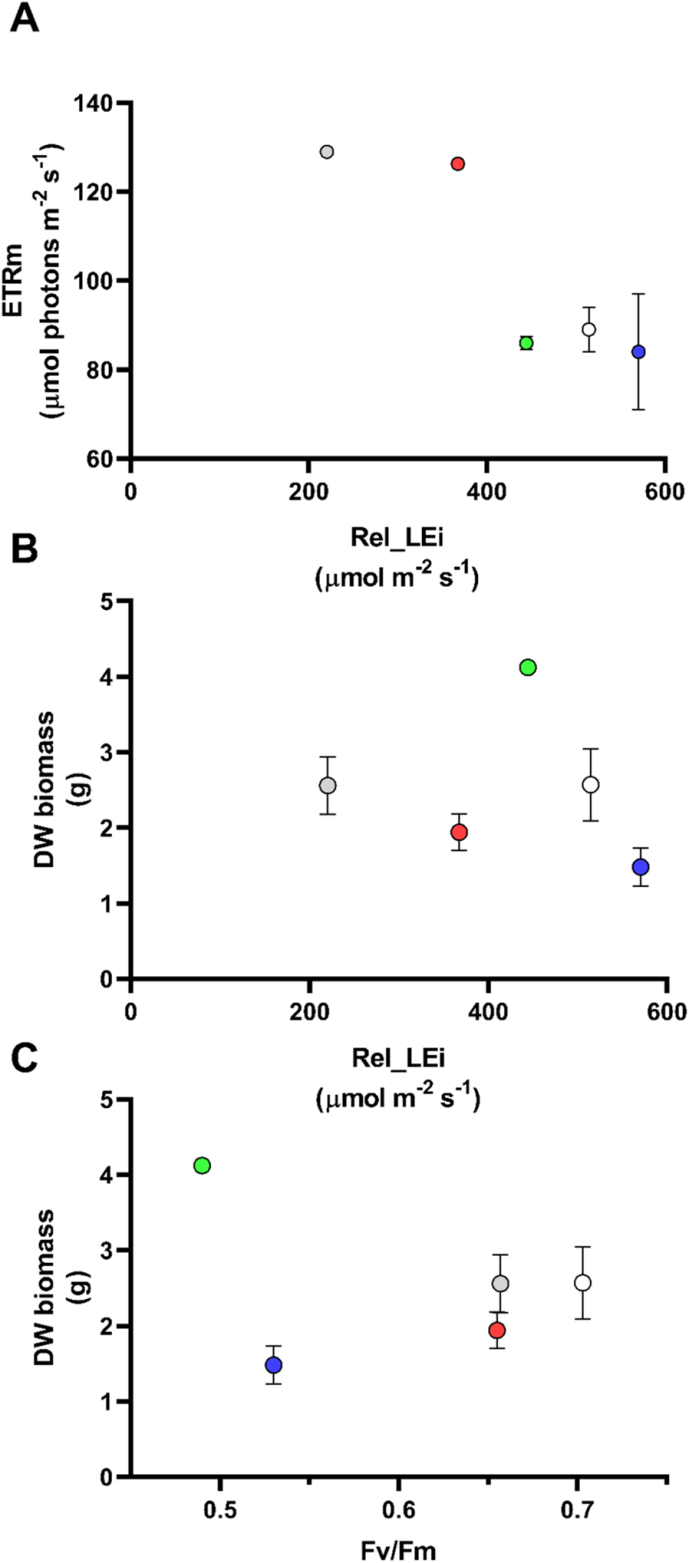
**A** Relative Electron transport rate (relETRm, μmol photons m^−2^ s^−1^) vs rel_LEi (Relative Light energy index; µmol m^−2^ s^−1^); **B** dried biomass (g DW) vs rel _LEi (µmol m^−2^ s^−1^); **C** Dried biomass (g DW) vs *F*_v_/*F*_m_. Blue (BHL condition); Red (RHL condition); Green (GHL condition); White (WHL condition); grey (WLL condition). See Table 2 for rel_LEi information and calculation

After 21 days of cultivation, none of the cultures presented macronutrient (nitrogen or phosphate) depletion (Additional file 1: Table S1), allowing to exclude any nutrient full consumption-related physiological/biochemical stress in cells. Photosynthetic rate (Electron transport rate, relETRm) was dependent on the rel_LEi, being significantly lower (*p* < 0.001) under high Energy light climate (rel_LEi ≧ 440 µmol m^−2^ s^−1^; GHL, WHL, and BHL) compared to the two lowest energy light conditions (rel_LEi ≦ 440 µmol m^−2^ s^−1^; RHL and WLL) (Fig. 1A). Conversely, the rel_LEi did not directly modulate the harvested biomass quantity (g DW; *p* > 0.05, *n* = 15, Fig. 1B), which ranged between 1.55 and 2.57 g except the highest value under GHL condition (DW_green_ = 4.2 g). The unbalance between biomass synthesis and photosynthesis was confirmed by the two-shapes relationship between ETRm per biomass unit (g DW^−1^) and rel_LEi (Additional file 2: Fig. S1A), presuming two different physiological states modulating the photosynthetic capacity per biomass unit below or above 440 µmol m^−2^ s^−1^. This feature was confirmed by the relationship between ETRm and the harvested biomass (g DW; Additional file 2: Fig. S1B) identifying the discrimination of the two low and high rel_LEi clusters. Conversely, DW was correlated with *F*_v_/*F*_m_ when excluding the outlier value measured under GHL condition (*p* < 0.05, *n* = 12; Fig. 1C).

**Fig. 2:**
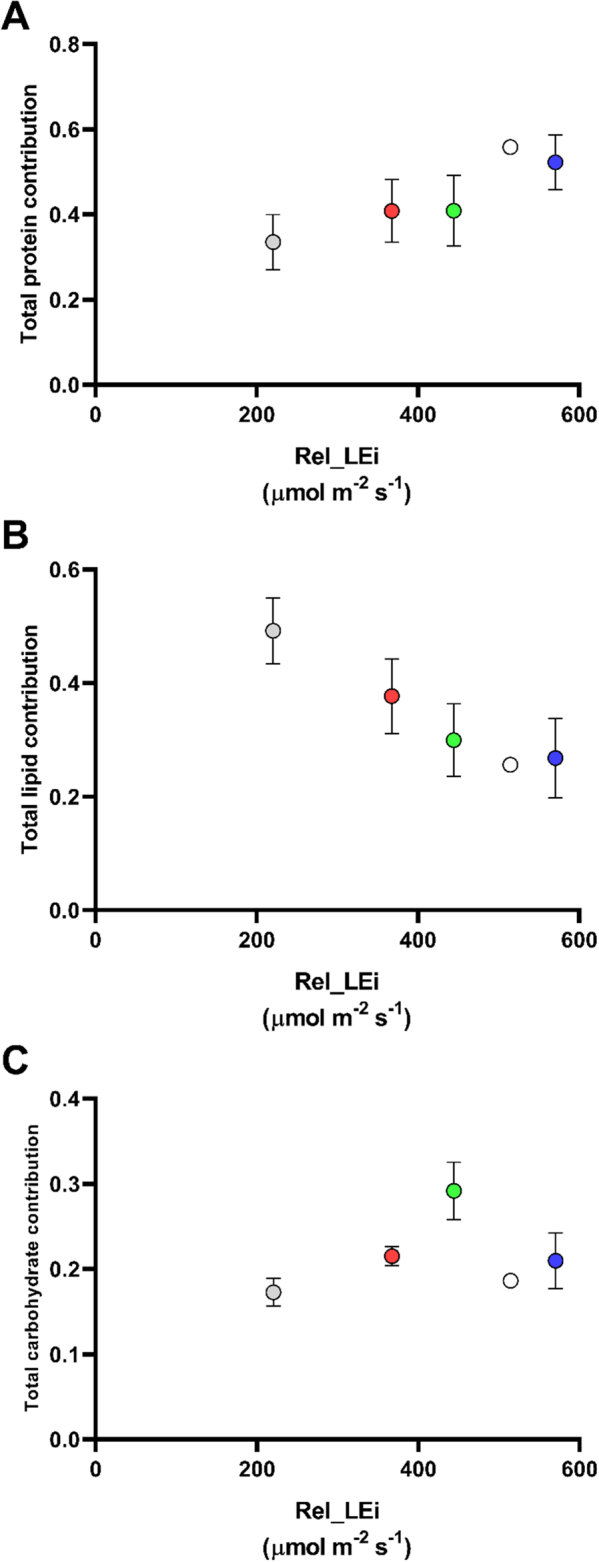
**A** Total protein contribution (protein vs sum of protein + carbohydrate + lipid) in the *S. subsalsa* biomass vs rel_LEi (µmol m^−2^ s^−1^); **B** total lipid contribution (lipid vs sum of protein + carbohydrate + lipid) in the *S. subsalsa* biomass vs rel_LEi (µmol m^−2^ s^−1^); **C** total carbohydrate contribution (carbohydrate vs sum of protein + carbohydrate + lipid) in the *S. subsalsa* biomass vs rel_LEi (µmol m^−2^ s^−1^). Blue (BHL condition); Red (RHL condition); Green (GHL condition); White (WHL condition); grey (WLL condition). See Table 2 for rel_LEi information and calculation

With respect to the macromolecular composition of the biomass, *Spirulina subsalsa* displayed a higher content of proteins (0.378 mg mgDW^−1^ ± 0.17) and lipids (0.313 mg mgDW^−1^ ± 0.08) compared to carbohydrates (0.175 mg mgDW^−1^ ± 0.048; *p* < 0.01). Total protein contribution (= protein vs sum of protein + carbohydrate + lipid) significantly increased with light energy (*p* < 0.01; *n* = 15) conversely to the total lipid contribution (= lipid *vs* sum of protein + carbohydrate + lipid) which displayed the inverse trend (*p* < 0.01; *n* = 15, Fig. 2A, B). Contribution of total carbohydrate (carbohydrate vs sum of protein + carbohydrate + lipid) was not linearly related to rel_LEi, but displayed two trends, below and above the rel_LEi value ~ 444 µmol m^−2^ s^−1^ (Fig. 2C).

### Total sterols, polyphenols and flavonoids

**Fig. 3:**
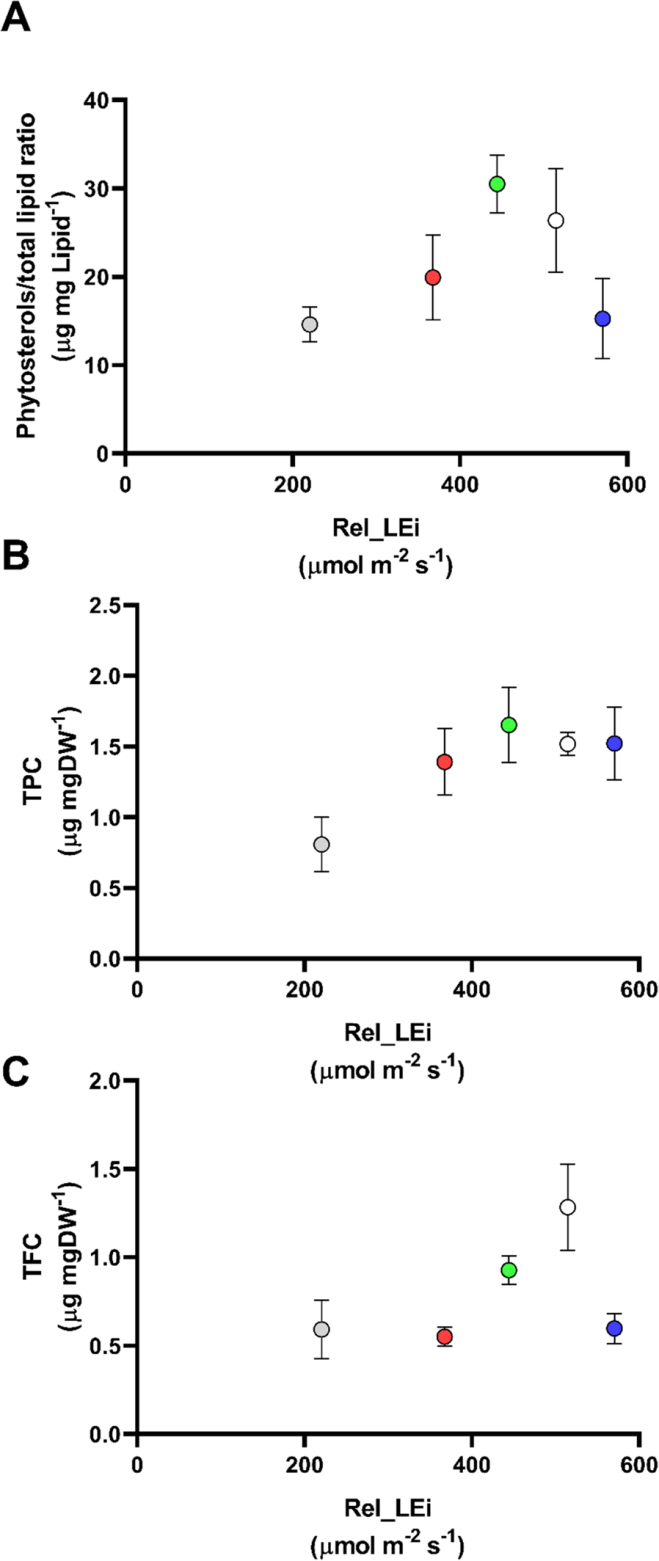
**A** Phytosterols*/*total lipid ratio (μg mg Lipid^−1^) in the *S. subsalsa* biomass *vs* rel_LEi (µmol m^−2^ s^−1^); **B** the total polyphenols content (TPC, μg mg DW^−1^) in the *S. subsalsa* biomass *vs* rel_LEi (µmol m^−2^ s^−1^); **C** the total flavonoids content (TFC, μg mg DW^−1^) in the *S. subsalsa* biomass vs rel_LEi (µmol m^−2^ s^−1^). Blue (BHL condition); Red (RHL condition); Green (GHL condition); White (WHL condition); grey (WLL condition). See Table 2 for rel_LEi information and calculation

The total phytosterol content (TSC) contributed to total lipids from 1.4% (± 0.2) to 3.1% (± 0.3). The phytosterol vs lipid ratio (μg mgLipid^−1^) displayed a hormetic curve increasing until a rel_LEi ~ 444 µmol m^−2^ s^−1^ followed then by a decrease at higher rel_LEi (Fig. 3A). This ratio was significantly related to the harvested biomass (gDW; *p* < 0.05; *n* = 15).

The total polyphenols content (TPC) was significantly correlated with rel_LEi (*p* < 0.02; *n* = 15) increasing with rel_LEi until to reach a plateau at rel_LEi ~ 444 µmol m^−2^ s^−1^ (Fig. 3B). Conversely, the total flavonoids content (TFC) did not follow any significant trend with rel_LEi (*p* > 0.05; *n* = 15; Fig. 3C).

Among the known phenolic compounds detected, apigenin was the most abundant (from 8 to 32 μg mgDW^−1^) followed by quercetin, gallic acid, rutin and cinnamic acid (Table 1). The content of the two compounds apigenin and cinnamic acid was significantly higher under WLL condition—the lowest light energy—compared to the other conditions (at least *p* < 0.02). By contrast, quercetin was slightly higher under WHL. The compounds catechin (flavonol) and *p*-coumaric acid (hydroxycinnamic acid) were only present under the lowest rel_LEi (WLL condition; Table 1), while the genistein (isoflavone) was only synthetized under the highest rel_LEi (BHL condition; Table 1).

**Table 1:** Phenolic compounds concentration (μg mgDW^−1^) in the *S. subsalsa* biomass grown under the different light climates

	Gallic acid	Catechin	*p*-Coumaric acid	Rutin	Cinnamic acid	Apigenin	Genistein	Quercetin
WLL	0.027 ± 0.0001	0.092 ± 0.023	0.004 ± 0.0005	0.033 ± 0.007	0.020 ± 0.001	32.236 ± 2.05	0.000	0.035 ± 0.008
WHL	0.035 ± 0.0028	0.000	0.000	0.022 ± 0.0058	0.007 ± 0.0007	0.000	0.000	0.040 ± 0.004
BHL	0.014 ± 0.0014	0.000	0.000	0.022 ± 0.0062	0.011 ± 0.0022	16.840 ± 1.24	6.086 ± 0.99	0.036 ± 0.001
GHL	0.025 ± 0.0028	0.000	0.000	0.019 ± 0.0053	0.013 ± 0.003	21.371 ± 2.71	0.000	0.037 ± 0.009
RHL	0.024 ± 0.0019	0.000	0.000	0.017 ± 0.006	0.007 ± 0.0003	8.257 ± 0.034	0.000	0.035 ± 0.006

### Carotenoids and phycobiliproteins

**Fig. 4:**
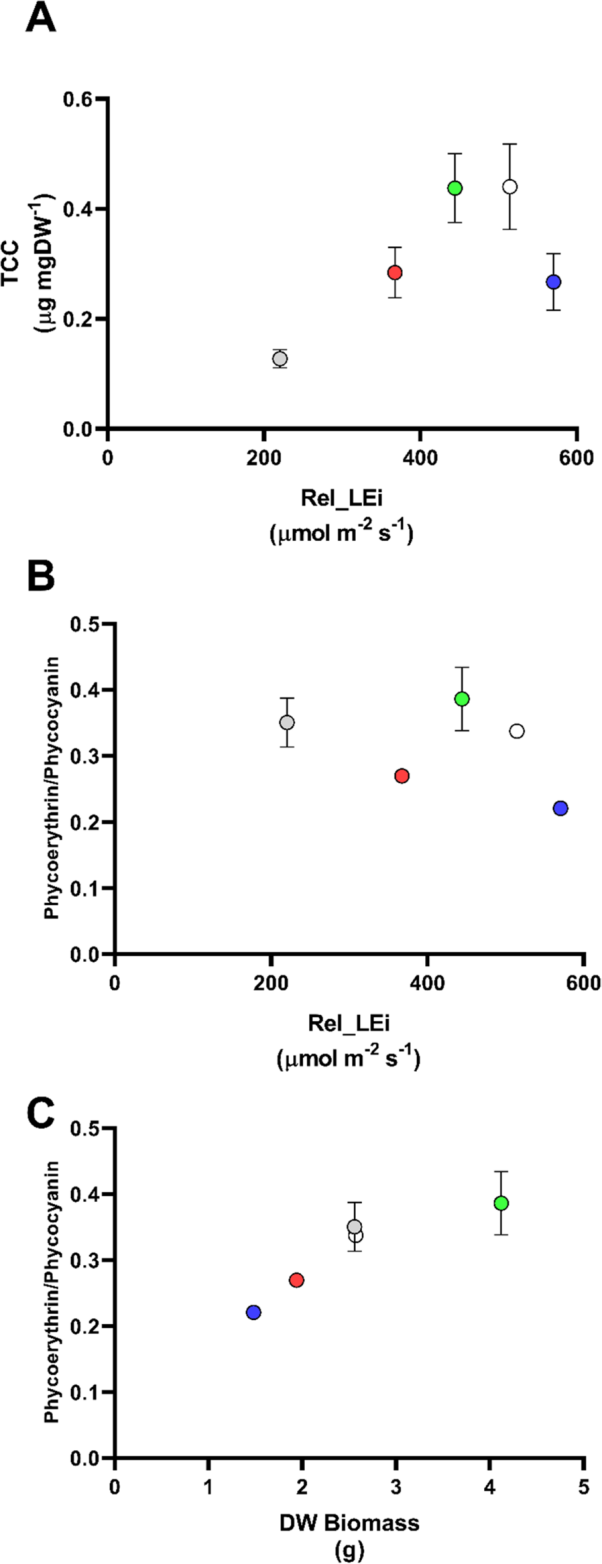
**A** Total carotenoid content (TCC, μg mg DW^−1^) in the *S. subsalsa* biomass vs rel_LEi (µmol m^−2^ s^−1^); **B** phycoerythrin/phycocyanin ratio (μg mg DW^−1^ vs μg mgDW^−1^) in the *S. subsalsa* biomass vs rel_LEi (µmol m^−2^ s^−1^); **C** phycoerythrin/phycocyanin ratio (μg mg DW^−1^ vs μg mg DW^−1^) in the *S. subsalsa* biomass vs DW biomass (g). Blue (BHL condition); Red (RHL condition); Green (GHL condition); White (WHL condition); grey (WLL condition). See Table 2 for rel_LEi information and calculation

The total carotenoid content (TCC) displayed an increasing trend over the rel_LEi gradient (*p* < 0.05, *n* = 15; Fig. 4A) with a high significant relationship (*p* < 0.001, *n* = 12) when excluding the BHL condition (highest rel_LEi) in which TCC decreased. Although the carotenoids hold an antioxidant activity—explaining their increase with rel_Lei—their maximal absorbance in the blue spectrum probably led to the decrease of their content to limit damages or because their use as antioxidant when blue PFD was too high. The two main carotenoids, zeaxanthin, and β-carotene, as well as the 4 keto-myxoxanthophyll followed the same trend as TCC (*p* < 0.001, *n* = 15), lowering their content under the highest rel_LEi (Additional file 3: Fig. S2A–C). By contrast, echinenone significantly increased with rel_LEi (*p* < 0.001, *n* = 15; Additional file 3: Fig. S2D). In addition, the carotenoids myxoxanthophyll and cryptoxanthin tended to increase with rel_LEi, excepting the low values reported under GHL and BHL, i.e., when the green (under BHL) or blue (under GHL) part of the spectrum was absent of the light climate.

Phycobiliproteins were dominated by phycocyanin (PC) compared to phycoerythrin (PE). Both PE and PC had a similar distribution, with highest values under WHL (only significant for PE, *p* < 0.05) and without any clear trend with rel_LEi (data not shown). The ratio PE/PC was the lowest under BHL and RHL, revealing the modulative impact of spectral properties, while no clear trend with rel_LEi was noticed (Fig. 4B). However, PE/PC was significantly correlated with DW biomass (*p* < 0.001; *n* = 15; Fig. 4C) and *F*_v_/*F*_m_, the latter when excluding the GHL values (*p* < 0.05; *n* = 12; data not shown).

### Vitamins

**Fig. 5:**
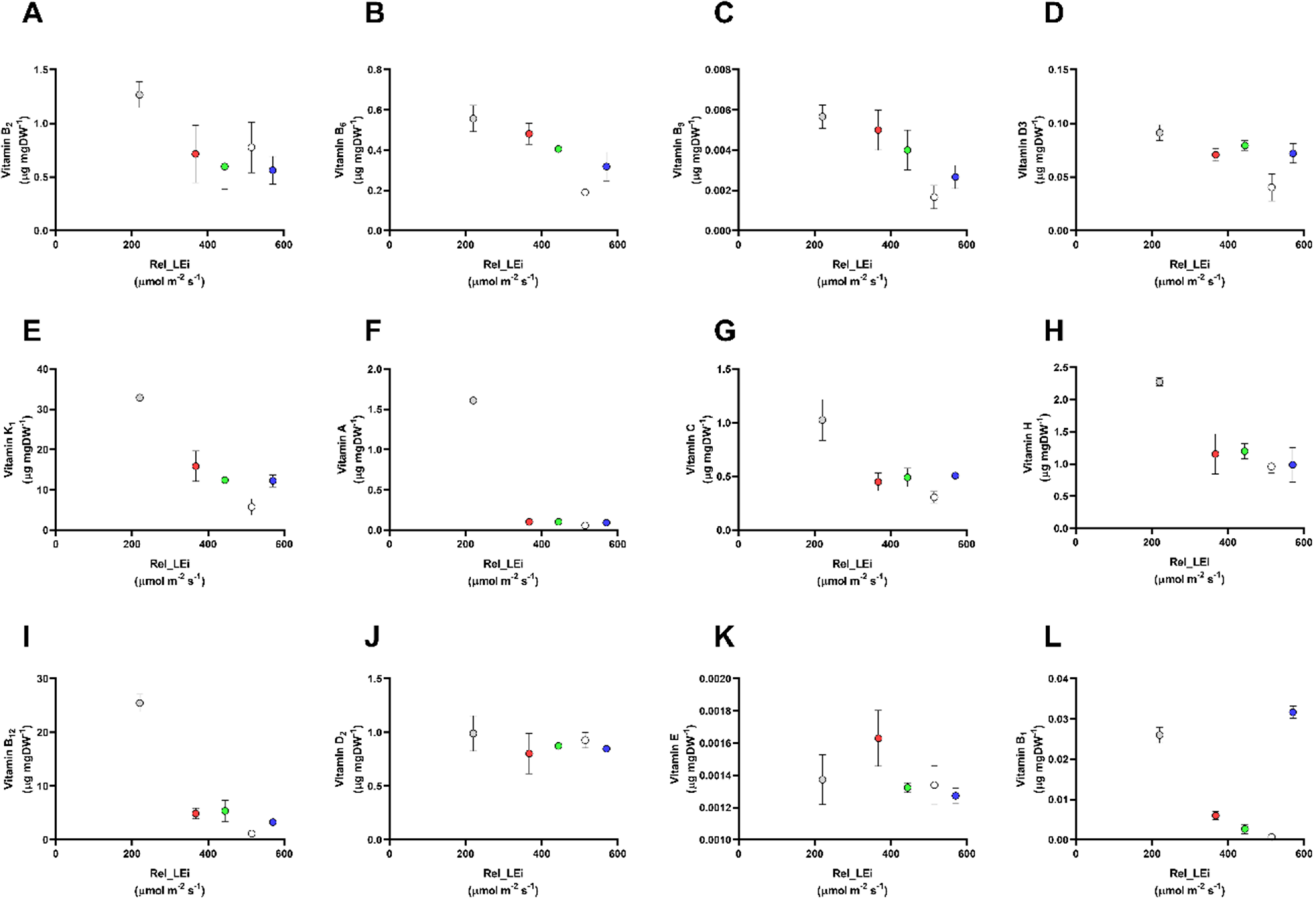
Vitamin concentrations in the *S. subsalsa* biomass vs rel_LEi (µmol m^−2^ s^−1^). **A** Vitamin B_2_ (μg mg DW^−1^); **B** vitamin B_6_ (μg mg DW^−1^); **C** vitamin B_9_ (μg mg DW^−1^); **D** vitamin D_3_ (μg mg DW^−1^); **E** vitamin K_1_ (μg mg DW^−1^); **F** vitamin A (μg mg DW^−1^); **G** vitamin C (μg mg DW^−1^); **H** vitamin H (μg mg DW^−1^); (I) vitamin B_12_ (μg mg DW^−1^); **J** vitamin D_2_ (μg mg DW^−1^); **K** vitamin E (μg mg DW^−1^); **L** vitamin B_1_ (μg mg DW^−1^). Blue (BHL condition); Red (RHL condition); Green (GHL condition); White (WHL condition); grey (WLL condition). See Table 2 for rel_LEi information and calculation

The 12 vitamins, water-soluble (B_1_, B_2_, B_6_, B_9_, B_12_, C, H) and fat-soluble (A, D_2_, D_3_, E, K_1_), displayed a broad range of concentrations, ranging from 0.4 ng mgDW^−1^ to 33 μg mgDW^−1^ (Fig. 5). Among these, the nine vitamins B_2_, B_6_, B_9_, D_3_, K_1_, A, C, H, and B_12_ had the highest content under the lowest rel_LEi (Fig. 5A–I) displaying a significantly inverse relationship with rel_LEi (at least *p* < 0.01 except for the vitamin C with *p* < 0.05; *n* = 15). Conversely, the vitamins D_2_ and E did not present any relevant variations among the light climates (Fig. 5J, K), while the vitamin B_1_ was significantly higher (*p* < 0.05) under the lowest and highest rel_LEi compared to the three other light climates (Fig. 5L).

### Antioxidant capacity

**Fig. 6:**
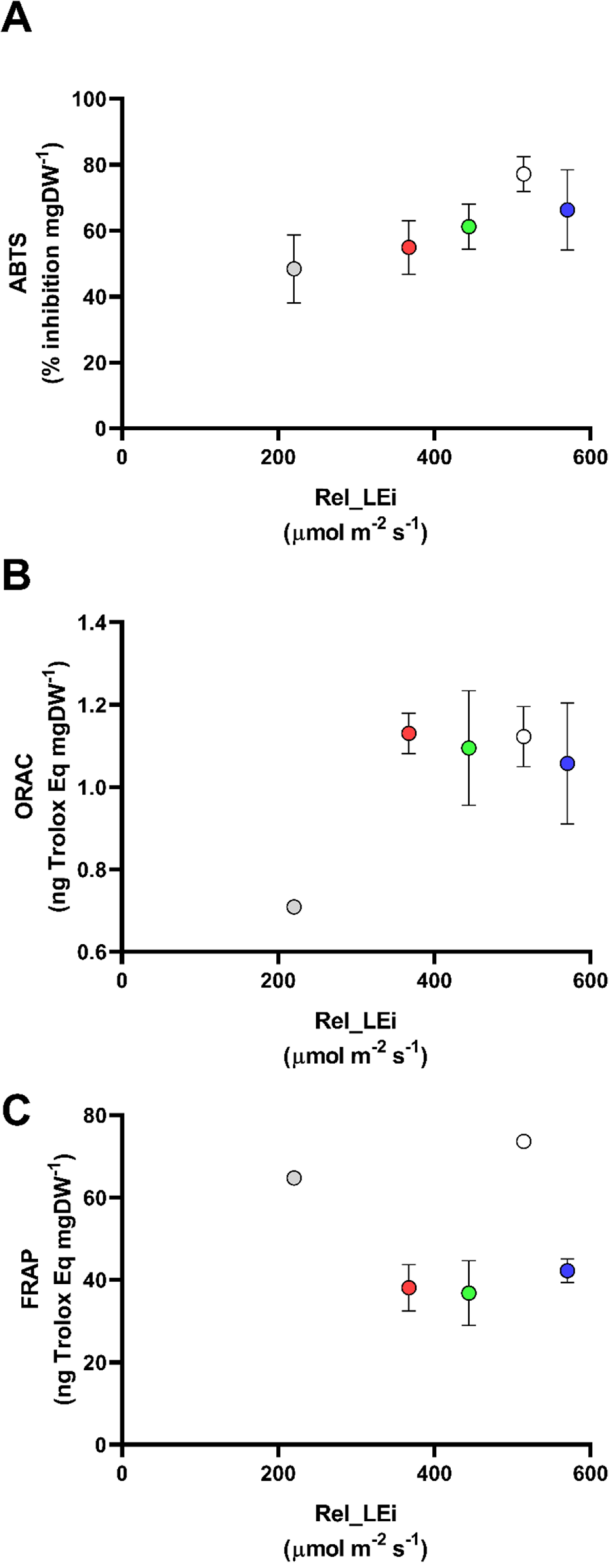
Antioxidant capacity of the biomass vs rel_LEi (µmol m^−2^ s^−1^). **A** Antioxidant capacity measured with the ABTS assay (% inhibition mg DW^−1^), **B** antioxidant capacity measured with the ORAC assay (ng of Trolox Equivalent mg DW^−1^), **C** antioxidant capacity measured with the FRAP assay (ng of Trolox Equivalent mg DW^−1^). Blue (BHL condition); Red (RHL condition); Green (GHL condition); White (WHL condition); grey (WLL condition). See Table 2 for rel_LEi information and calculation

The antioxidant capacity of the biomass estimated with ABTS assay was significantly correlated with the rel_LEi (*p* < 0.01, *n* = 15) presenting the same trend than TCC (Fig. 6A), i.e., with a small decrease of the values under the highest rel_LEi (BHL condition). Indeed, ABTS was significantly correlated with TCC (*p* < 0.05, *n* = 15). The ORAC-related antioxidant property (Fig. 6B) was significantly correlated with the ABTS-related antioxidant property (*p* < 0.05, *n* = 15) as well as to the TCC (*p* < 0.05, *n* = 15) but not to the rel_LEi (*p* > 0.05, *n* = 15). Conversely, the FRAP-related antioxidant property did not show any dependency on light energy (*p* > 0.05, *n* = 15; Fig. 6C), with the highest values reported under low (WLL) and high (WHL) white lights. FRAP was significantly correlated with PE (*p* < 0.01, *n* = 15) and PC (*p* < 0.05, *n* = 15).

### Principal component analysis

**Fig. 7:**
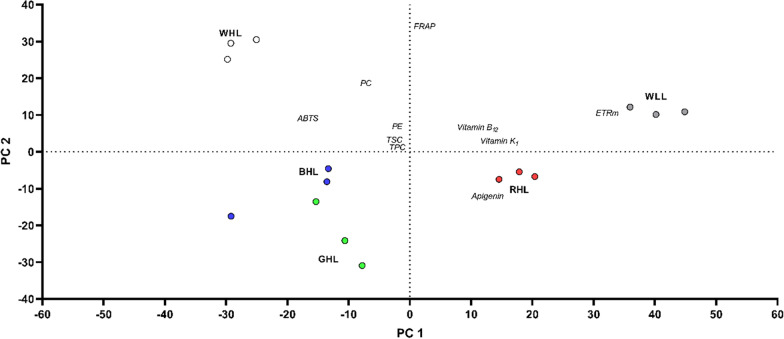
Bi-plot of the Principal Component Analysis (PCA). The axes 1 and 2 explained 53% and 27% of the total variance, respectively. The PCA was performed on the variance–covariance matrix including the photosynthetic/photo-acclimation properties, macromolecular composition, bioactive compounds and antioxidant capacities of *Spirulina subsala* grown under the five light conditions

The two first axes of the principal component analysis did account for 80% of the variance (53% and 27% for axis 1 and 2, respectively). The bi-plot (Fig. 7) reported an opposite distribution of the low (WLL and RHL) and high rel_LEi (WHL, BHL, GHL) along the axis 1. The low light energy cluster (WLL and RHL) was related to the variables relETRm, vitamin content (particularly, K_1_ and B_12_) and apigenin; the variables ABTS, TPC and PC were instead directed towards the high light energy cluster (WHL, BHL and GHL). The axis 2 discriminated the spectrally balanced light climates (WHL and WLL, upper part of the axis 2) from the spectrally unbalanced light climates (RHL, BHL and GHL; lower part of the axis 2). The variables FRAP, PE and PC were opposed to the spectrally unbalanced light climates.

## Discussion

Our study explores a *Biotechnological Response Curve* design contemplating bioactive secondary metabolites synthesis modulation over a light energy gradient. The relative Energy light index, based on the PFD of a given part of the spectrum and on the relative energy (electron volt) associated with this spectrum, gives functional insights on the role of light in modulating microalgal metabolism. Indeed, it integrates in one dimension the multi-factorial nature of light (spectrum, energy and PFD) providing a clearer understanding of the biological response to light variations. This is the case for the antioxidant properties of the biomass, which strongly relies to the rel_LEi index. The ABTS vs rel_LEi relationship depicts features that remain hidden when using other light variables (comparing the Fig. 6A with the Additional file 4: Fig. S3), revealing a role of energy—beyond the spectral contribution and intensity roles—on the physiological state and photosystem functioning.

Results reveal the succession of low light adapted and high light adapted *Spirulina subsalsa,* with a threshold between the two states at a rel_LEi ~ 400 μmol m^−2^ s^−1,^ i.e., a mid-day PFD peak of 350 μmol m^−2^ s^−1^ dominated by green spectrum. As comparative exercise, a mid-day PFD peak of 350 μmol m^−2^ s^−1^ composed by a solar-type spectrum (similar contribution of red, green and blue) leads to a rel_LEi ~ 466 μmol m^−2^ s^−1^, while a monochromatic blue light provides a rel_LEi ~ 600 or 250 μmol m^−2^ s^−1^ when setup at PFD of 350 or 150 μmol m^−2^ s^−1^, respectively.

The boundary between low light response *vs* high light response represented by the mid-day PFD peak of 350 μmol m^−2^ s^−1^ reveals that *S. subsalsa* is a low or moderate light acclimated species, such as the close species *Spirulina maxima* or *platensis* are [27–30]. Indeed, our experimental setup reproduces a natural light distribution following a sinusoidal curve during the daylight period, which almost halves the daily light dose compared to a quadratic continuous light distribution over the 12 h of illumination [31].

The hormetic curve of the phytosterol *vs* lipid content confirms the two physiological states succession, as well as the relevant role of light in modulating phytosterols content in microalgae [32–36], modulating the membrane fluidity and permeability [37]. High light intensity, as well as blue light (= high light energy), are known to decrease the phytosterol content in microalgae [32, 34–36]. The documented phytosterol content decrease belongs to the high light energy-induced cascade of responses, together with the lowering of photosynthesis and increase in protective and defense requirements. The high light regulation is characterized by an increase of the antioxidant property (ABTS, ORAC) of the biomass, related to the rise in total carotenoids and polyphenols, well-known antioxidant families. However, the single carotenoids do not similarly respond to light energy gradient, due to the differences in their localization or role [38]. For instance, myxoxanthophyll is involved in thylakoid membrane rigidity [38], while echinenone is more abundant in cytoplasmic membranes than in thylakoids [38]. Nonetheless, the diverse antioxidant ability or bioactive properties of carotenoids might also explain their diverse behavior, especially under the highest light energy [39].

The response of phenolic compounds to the light energy gradient is more variable than for carotenoids, in agreement with the high chemodiversity of these compounds [7] and with their complex and diverse biosynthetic pathways [40]. Only the isoflavone genistein and the flavonoid quercetin tended to be higher under high light energy conditions, a feature that well fits the potent antioxidant activity of these two polyphenols [41, 42].

By contrast to other bioactive compound families, the lower content of many vitamins (B_2_, B_6_, B_9_, D_3_, K_1_, A, C, H and B_12_) under high light energy suggests that cells might use them as cofactors to perform high light energy-driven biological functions. Indeed, many vitamins (e.g., A, C, B_6_, D_3_) are well-known antioxidants [6, 43–46], while many vitamins B are used as co-factors or substrate for a plethora of enzymes [47–49]. Although the other vitamins, D_2_, B_1_ and E are not consumed under high energy or are rapidly renewed, our results suggest that light energy manipulation induces a divarication between biomass with higher content of carotenoids, polyphenols or biomass with higher content in many vitamins.

Another noteworthy result is the divergence between the ABTS and ORAC assays on one hand and the FRAP test on the other hand [50–54]. ABTS assay is connected with both the lipophilic compounds, such as carotenoids, and the lipophilic–hydrophilic phenolic compounds [54, this study], resulting in a highly responsive antioxidant test, as also confirmed by the significant relationship with light energy. Conversely, ORAC assay is only connected with the lipophilic carotenoids, while the FRAP assay does only respond to the hydrophilic phycobiliproteins (PE or PC) content. The depicted relationships between these antioxidant assays and the chemical properties of bioactive compound families highlight their complementarity and the need of combined use in bioprospecting issue.

## Conclusions

The relative light energy index (rel_LEi) is a proxy combining the PAR photon flux density data with the relative contribution of the different wavelengths. Further studies are needed to deeply analyze the appropriateness and limitations of rel_LEi. For instance, the relative light energy index might vary with different spectral (R, G, or B) contribution in a determined PAR or with a determined spectral (R, G, or B) contribution into a varying PAR. The effects of these two properties on the biological/physiological properties of the microalga have to be investigated at a functional point of view. Once the limitations of such approach defined, the light energy index can be also employed in microalgal productive plants to manipulate the synthesis of antioxidant families. Indeed, this exploratory investigation emphasizes the rel_LEi index as a relevant tool to explain light climate impacts on biological responses in microalgae. Our study reveals that this is still more important for the exploration of secondary metabolites synthesis, such as those displaying antioxidant role. We, therefore, propound that the *Biotechnological response curve* strategy using the light energy index might become a foundation in microalgal bioprospecting context endowing human health benefits.

## Materials and methods

### Experimental design and light climates

The cyanobacterium *Spirulina subsalsa* (CCMP796) was grown at 20 °C in 5 L parallelepiped glass containers in autoclaved seawater, pre-filtered through a 0.7 μm GF/F glass–fiber filter and enriched with F/2 culture medium nutrients [55]. In the tank, water movement was carried out using an aquarium wave maker pump (Sunsun, JVP-110) [55]. During the experiments carried out in triplicate, growth of *S. subsalsa* lasted 21 days, with an initial concentration of the inoculum of 0.15 g L^−1^ of fresh *S. subsalsa* biomass.. Light intensity was measured inside each tank with a PAR 4π sensor (QSL 2101, Biospherical Instruments Inc., San Diego, CA, USA), while the spectral composition was measured using a spectroradiometer (Hyper OCR I, Satlantic, Halifax, CA, USA).

Five light climates were setup through a patented custom-built LED illumination system [12, 56]. All experimental light climates were characterized by a sinusoidal light distribution with a photoperiod of 12 h:12 h light:dark, while differed by the red:green:blue (R:G:B) balance (blue: 455 ± 30 nm; green: 530 ± 30 nm; red: 630 ± 30 nm), and/or by the mid-day photon flux density setup at 150 or 350 µmol m^−2^ s^−1^ (Table 2). Growth of cells was performed under a light climate characterized by a mid-day light peak intensity setup at 150 µmol m^−2^ s^−1^ with a R:G:B = 10:40:50. In the four high-light climate conditions (GHL, RHL, BHL, WHL; Table 2), at the dawn of day 20 of growth, i.e., the day before the sampling day, cells were shifted to conditions characterized by a mid-day light peak intensity of 350 µmol m^−2^ s^−1^ to boost the production of bioactive metabolites [54]. High light conditions were characterized by three unbalanced spectral conditions (R:G:B = 10:0:90, R:G:B = 10:90:0 and R:G:B = 90:5:5) together with a real in situ spectral composition (R:G:B = 10:40:50) [12, 31, 57]. In each of the three unnatural spectral conditions, one the three wavelengths (R, G, or B) was setup at 90% (315 µmol m^−2^ s^−1^). When red flux density was dominating, blue and green spectra were provided together (5% each); while when blue or green photon flux density was dominating, the other one was absent to maintain the red contribution at 10% like in WHL and WLL (Table 2). Maintaining a stable red contribution (35 µmol m^−2^ s^−1^ in case of HL conditions) prevented introducing a new photoreceptor-dependent variability.

**Table 2:** Properties of the five light climates

					Energy (eV)	rel_eV_λ_
				Red	1.8	1
				Green	2.33	1.3
				Blue	3.1	1.7

	PFD (μmol m^−2^ s^−1^)	R:G:B (%)	PFD_Red_ (μmol m^−2^ s^−1^)	PFD_Green_ (μmol m^−2^ s^−1^)	PFD_Blue_ (μmol m^−2^ s^−1^)	rel_Lei (μmol m^−2^ s^−1^)
WLL	150	10:40:50	15	60	75	220.5
WHL	350	10:40:50	35	140	175	514.5
BHL	350	10:00:90	35	0	315	570.5
GHL	350	10:90:00	35	315	0	444.5
RHL	350	90:05:05	315	17.5	17.5	367.5

R:G:B: red:green:blue; PFD: photon flux density at the mid-day peak (μmol m^−2^ s^−1^); rel_LEi: relative light energy index (in µmol m^−2^ s^−1^; see text); rel_eV_λ_: relative energy in electron volt of the red, blue or green photon (see text)

To combine photon flux density (PFD) and the light spectral composition, we defined a relative Light energy index (rel_LEi in µmol m^−2^ s^−1^, Table 2). This index was calculated as follows:

$$ {\text{rel}}\_{\text{LEi}} = \sum {({\text{PFD}}_{\lambda } \times {\text{rel}}\_{\text{eV}}_{\lambda } ),} $$

where PFD_*λ*_ was the photon flux density at mid-day (i.e., the PFD peak) of the red, blue or green wavelength and rel_eV_*λ*_ was the relative energy in electron volt of the red, blue or green wavelength. Rel_eV_*λ*_ was calculated applying a rel_eV_red_ = 1 (red has the lower energy, eV_red_ = 1.8), rel_eV_green_ = 1.3 (eV_green_/eV_red_ with eV_green_ = 2.33) and rel_eV_blue_ = 1.7 (eV_blue_/eV_red_ with eV_blue_ = 3.10).

### Sampling

Sampling was carried out in the dark, i.e., before dawn at the day 21 of growth. Cells were collected in sterile condition onto a plankton net piece (mesh size 60 µm) to harvest biomass. Cells were then harvested and centrifuged at 2000×*g* for 15 min at 4 °C (DR15P centrifuge, B. Braun Biotech International, Melsungen, Germany). Pellets were flash-frozen in liquid nitrogen and stored at − 20 °C. Pellet was subsequently lyophilized in a Freeze Dryer Modulyo (Edwards LifeSciences, Irvine, CA, USA). Dry weight was accurately measured (dry weight, g DW).

### Photochemical efficiency of the photosystem II and electron transport rate–light curves

Photochemical Efficiency of the Photosystem II (*F*_v_/*F*_m_) was measured using a DUAL–PAM fluorometer (Heinz Walz GmbH, Effeltrich, Germany) in dark-acclimated samples.

The electron transport rate (ETR) vs irradiance (*E*) curves were determined on 15-min dark-acclimated samples by applying a series of nine increasing actinic light intensities (composed by 2/3 of blue and 1/3 of red light, lasting 1.0 min each, ranging from 1 to 1222 μmol photons m^−2^ s^−1^). The protocol was the same reported by Smerilli et al. [58].

The relative ETR, was calculated as follows:

$$ {\text{relETR}} = F^{\prime}_{{\text{v}}} /F^{\prime}_{{\text{m}}} \cdot E \cdot 0.5, $$

where *E* was irradiance, 0.5 was applied assuming that half of the incident light was absorbed by the PSI and half by the PSII. The relative ETR was expressed in μmol photons m^−2^ s^−1^ and then normalized on g of DW (μmol m^−2^ s^−1^ gDW^−1^). Determination of the photosynthetic parameters (relETRmax, Ek) was retrieved according to the equation of Eilers and Peeters [59].

### Total carbohydrate content

Total carbohydrate concentration was determined on 30 mg of dried algal powder with the phenol sulfuric acid method [60], slightly modified as described by Pistelli et al. [8].

### Total protein content

Total protein content was estimated on aliquots of 30 mg of dried microalgal powder re-suspended in 500 μL of RIPA (Radio-Immunoprecipitation Assay) and Extraction Buffer (Thermo Fisher Scientific, Waltham, MA, USA) and sonicated for 90 s (30 s on, 30 s off, 30 s on) using a micro tip at 20% output on ice (S-250A Branson Ultrasonic). Samples were then centrifuged at 13,000×*g* for 4 min, and supernatants were collected for protein content estimation. For this, 20 μL of sample were added into a 96 well plate (transparent flat bottom, TPP Techno Plastic Products AG, Trasadingen, Switzerland), with then the addition of 200 μL of Bradford reagent (A6932, AppliChem GmbH, Darmstadt, Germany). The absorbance was read at 595 nm, using a Microplate Reader: Infinite® M1000 PRO (TECAN, Männedorf, Switzerland). The total protein concentration was quantified referring to a calibration curve using bovine serum albumin (BSA) as standard.

### Total lipid and sterol content

Total lipid concentration was estimated on aliquots of 30 mg of dried microalgal powder, applying the method previously described by Pistelli et al. [8]. The dried total lipid content was then used for total sterol concentration (TSC) estimation, following the method of Araújo et al. [61].

### Total polyphenol content and total flavonoid content

The total phenolic content (TPC) and total flavonoid content (TFC) of the microalgal biomass were estimated on aliquots of 10 mg of dried powder, performing Folin-Ciocalteu’s and aluminium chloride (AlCl_3_) colorimetric methods for TPC and TFC, respectively. The assays were performed following the methods described by Smerilli et al. [54].

### Phenolic compounds

Phenolic compound analysis was conducted by High Performance Liquid Chromatography (HPLC) analysis as described by Pistelli et al. [8]. Pure standards, purchased from the Merck KGaA (Darmstadt, Germany) were used to allow the quantification of phenolic compounds.

### Vitamins

Dried microalgal powder (15 mg) was used for a competitive ELISA (enzyme-linked immunosorbent assay (ELISA) Assay using specific antibodies to determine the content of the twelve vitamins, namely, vitamins A, B_1_, B_2_, B_6_, B_9_, B_12_, C, D_2_, D_3_, E, H, and K_1_. The assay was performed following the method previously described by Pistelli et al. [8], using specific primary antibody for each vitamin (Additional file 5: Table S2). Quantification of vitamins were carried out thanks to a calibration curve obtained using pure vitamin standards.

### Carotenoids

Pigment analysis was conducted by HPLC on 10 mg DW samples, following the method described by Pistelli et al. [8]. Determination and quantification of pigments were carried out using pigment standards from the D.H.I. Water and Environment (Danish Hydraulic Institute; Horsholm, Denmark).

### Phycobiliproteins

Phycobiliprotein analysis was carried out on 10 mg DW samples. The protocol used was adapted from Lantoine and Neveux [62], with phycobiliproteins extraction done in 4 ml of 0.1 M phosphate buffer (pH 6.58) and sonicated for 1 min. Then, samples were filtered onto GF/F Filters (25 mm, Whatman, Maidstone, United Kingdom). Optical measurements were done in a RF-6000 spectrofluorometer (Shimadzu, Kyoto, Japan). Excitation and emission wavelengths of 547 and 572 nm, respectively, were applied for phycoerythrin (PE) determination, while 600 and 643 nm were used for phycocyanin (PC) determination. Quantification of PE and PC were carried out thanks to a calibration curve obtained using pure C-Phycocyanin (Cat. No. 52468, Sigma-Aldrich, St. Louis, MO, USA) and B-Phycoerythrin from *Porphyridium cruentum* (Cat. No. P1286, Sigma-Aldrich, St. Louis, MO, USA).

### Antioxidant power

The ABTS *(2,2′-Azinobis-3-Ethylbenzothiazoline-6-Sulphonic Acid)* assay was carried out using aliquots of 50 mg of dried algal powder. The assay is based on the ability of the extract to scavenge ABTS^+^ radical, following the protocol developed by Pistelli et al. [8].

The ORAC assay *(Oxygen Radical Absorbance Capacity)* was carried out using aliquots of 10 mg of dried algal powder. Microalgal dry biomass was re-suspended in 2 mL of methanol and shacked vigorously for 2 min. The samples were centrifuged at 13,000×*g* for 10 min at 4 °C and then the supernatants were transferred into fresh tubes. The assay was performed following the method described by Kenny et al. [52]. Briefly, 100 μL of each sample was mixed with 600 μL of fluorescein solution and incubated for 10 min at 37 °C and dark. Fluorescein solution (final concentration of 81.6 nM) was prepared dissolving the fluorescein salt (C_20_H_10_Na_2_O_5_) in phosphate buffer (75 mM, pH 7.4). Then, 100 μL of AAPH ((2,2′-Azobis(2-methylpropionamide)) solution were added to each tube, and 125 μL of this solution were transferred to a black flat-bottom 96-well microplate (Corning Incorporated, Corning, NY, USA). The AAPH solution (153 mM) was prepared by dissolving 415 mg of AAPH dihydrochloride (C_8_H_20_Cl_2_N_6_) in 10 mL of phosphate buffer. Finally, the fluorescence was measured at 515 nm, using Infinite® M1000 PRO (Ex: 495 nm, Em: 515 nm; TECAN, Männedorf, Switzerland), after incubating for 35 min at dark and room temperature. The calibration curve was setup using the (±)-6-hydroxy-2,5,7,8 tetramethylchromane-2-carboxylic acid (Trolox) as standard. Trolox powder (CAS No. 53188-07-1, Cat. No. 238813, Sigma-Aldrich, St. Louis, MO, USA) was weighted and dissolved in methanol to obtain six concentrations, namely, 50, 40, 30, 20, 10, and 5 μM. Results were expressed as ng of Trolox Equivalent (TE).

The FRAP (*Ferric Reducing Antioxidant Power*) was carried out using aliquots of 10 mg of dried algal powder. Microalgal dry biomass was re-suspended in 2 mL of methanol and shacked vigorously for 2 min. The samples were centrifuged at 13,000×*g* for 10 min at 4 °C and then the supernatants were transferred into fresh tubes. The assay was performed following the method described by Kenny et al. [51]. A working FRAP solution of 100 mL of acetate buffer (300 mM, pH 3.6), 10 mL of FeCl_3_ (20 mM) and 10 mL of 2,4,6-Tri(2-pyridyl)-s-triazine (TPTZ, 10 mM) in HCl (40 mM) was prepared and incubated at 37 °C for 10 min at dark. For the assay, 20 μL of each sample and 180 μL of FRAP working solution were mixed in a 96 well plate, transparent flat bottom (TPP Techno Plastic Products AG, Trasadingen, Switzerland). The plate was incubated for 40 min at 37 °C at dark. After incubation, the absorbance was measured at 593 nm, using Infinite® M1000 PRO (TECAN, Männedorf, Switzerland). The calibration curve was setup using the (±)-6-hydroxy-2,5,7,8-tetramethylchromane-2-carboxylic acid (Trolox) as standard. Trolox powder (CAS No. 53188-07-1, Cat. No. 238813, Sigma-Aldrich, St. Louis, MO, USA) was weighted and dissolved in methanol to obtain six concentrations, namely, 480, 400, 320, 240, 160, and 80 μM. Results are expressed as ng of Trolox Equivalent (TE).

### Nutrient concentrations

Culture medium samples for the determination of nutrient concentrations were collected in 20 mL polyethylene vials. Ammonium, nitrate, nitrite, and phosphate concentrations were determined using a Technicon Auto Analyzer following classical method [63].

### Statistical analysis

All experiments were performed in triplicate and mean ± standard deviation (SD) was calculated using GraphPad Prism 8.0. One-way analysis of variance (ANOVA) followed by Tukey’s post hoc test were calculated using GraphPad Prism 8.0. Spearman correlation between the different variables (all data, *n* = 15) as well as Student’s *t* test and Mann–Whitney test were carried out using the PAST software package, version 3.10 [64]. Principal components analysis (PCA) on variance–covariance matrix was performed using the PAST software package, version 3.10 [64], including the photosynthetic/photo-acclimation properties, macromolecular composition, bioactive compounds and antioxidant capacities of the *Spirulina subsala* grown under the different five light conditions.

## Supplementary Information

**Additional file 1: Table S1.** Concentration of macronutrients in the culture medium after the 21 days of cultivation.

**Additional file 2: Figure S1.** (A) Relative Electron transport rate (relETRm)/DW biomass (μmol m^−2^ s^−1^ g DW^−1^) vs rel_LEi (µmol m^−2^ s^−1^); (B) relative Electron transport rate (relETRm, μmol m^−2^ s^−1^) vs DW biomass (g). Blue (BHL condition); Red (RHL condition); Green (GHL condition); White (WHL condition); grey (WLL condition). See Table 2 for rel_LEi information and calculation.

**Additional file 3: Figure S2.** Carotenoid concentration in the *S. subsala* biomass vs rel_LEi (µmol m^−2^ s^−1^). (A) Zeaxanthin (μg mg DW^−1^); (B) β-carotene (μg mg DW^−1^); (C) 4 keto-myxoxanthophyll (μg mg DW^−1^); (D) echinenone (μg mg DW^−1^); (E) myxoxanthophyll (μg mg DW^−1^); (F) cryptoxanthin (μg mg DW^−1^). Blue (BHL condition); Red (RHL condition); Green (GHL condition); White (WHL condition); grey (WLL condition). See Table 2 for rel_LEi information and calculation.

**Additional file 4: Figure S3.** Comparative distribution of the antioxidant property of the *S. subsala* biomass estimated with ABTS assay (% inhibition mgDW^−1^) vs different light indexes characterizing the five experimental light climates (see Table 2 and Fig. 6A): (A) ABTS vs PFD at light peak (μmol m^−2^ s^−1^); (B) ABTS *vs* green spectrum PFD at light peak (μmol m^−2^ s^−1^); (C) ABTS vs red spectrum PFD at light peak (μmol m^−2^ s^−1^); (D) ABTS *vs* blue spectrum PFD at light peak (μmol m^−2^ s^−1^); (E) ABTS vs blue + green spectra PFD at light peak (μmol m^−2^ s^−1^); (F) ABTS *vs* red spectrum contribution (%); (G) ABTS *vs* green spectrum contribution (%); (H) ABTS *vs* blue spectrum contribution (%). Blue (BHL condition); Red (RHL condition); Green (GHL condition); White (WHL condition); grey (WLL condition). See Table 2 for rel_LEi information and calculation.

**Additional file 5: Table S2.** List of the twelve antibodies used for vitamin determination applying competitive ELISA assay.

## Data Availability

All data are available upon request to the corresponding author.
