# Supplementary material for: Biotechnological response curve of the cyanobacterium Spirulina subsalsa to light energy gradient
Source: Biotechnol Biofuels Bioprod. 2023 Feb 19;16:28. doi: 10.1186/s13068-023-02277-4 (PMC9940373; doi:10.1186/s13068-023-02277-4)
Supplement: Supplementary file 1 — Additional file 1: Table S1. Concentration of macronutrients in the culture medium after the 21 days of cultivation. [file 13068_2023_2277_MOESM1_ESM.docx]

**Table S1: Concentration of macronutrients in the culture medium after the 21 days of cultivation**

|  | Nitrate concentration  (µmol L^-1^) | Nitrite concentration  (µmol L^-1^) | Ammonium  Concentration  (µmol L^-1^) | Phosphate concentration  (µmol L^-1^) |
| --- | --- | --- | --- | --- |
| WLL | 454,70  ± 106,48 | 3,27  ± 1,07 | 372,04  ± 147,88 | 10,11  ± 0,13 |
| WHL | 232,44  ± 36,14 | 4,09  ± 0,66 | 148,75  ± 10,32 | 20,40  ± 1,89 |
| BHL | 396,85  ± 91,83 | 15,99  ± 1,39 | 496,85  ± 23,18 | 12,76  ± 1,52 |
| GHL | 269,80  ± 41,30 | 7,78  ± 1,26 | 155,25  ± 20,22 | 10,12  ± 1,06 |
| RHL | 488,39  ± 22,18 | 7,81  ± 1,89 | 528,04  ± 70,58 | 15,15  ± 1,12 |
