# Supplementary figures and images for: Biotechnological response curve of the cyanobacterium Spirulina subsalsa to light energy gradient
Source: Biotechnol Biofuels Bioprod. 2023 Feb 19;16:28. doi: 10.1186/s13068-023-02277-4 (PMC9940373; doi:10.1186/s13068-023-02277-4)

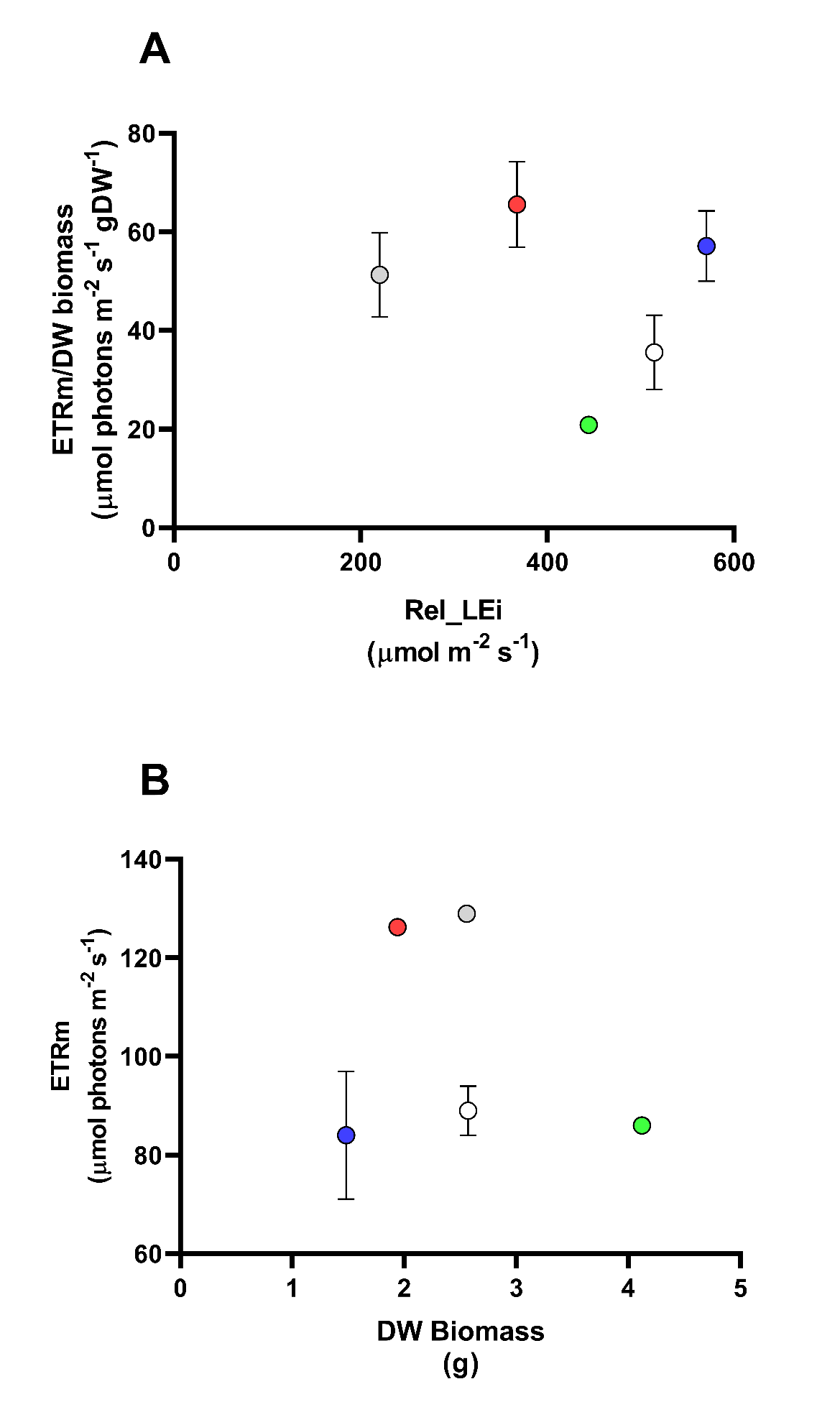


**Figure S1.**

Supplement: Supplementary file 2 — Additional file 2: Figure S1. (A) Relative Electron transport rate (relETRm)/DW biomass (μmol m−2 s−1 g DW−1) vs rel_LEi (µmol m−2 s−1); (B) relative Electron transport rate (relETRm, μmol m−2 s−1) vs DW biomass (g). Blue (BHL condition); Red (RHL condition); Green (GHL condition); White (WHL condition); grey (WLL condition). See Table 2 for rel_LEi information and calculation. [file 13068_2023_2277_MOESM2_ESM.docx]

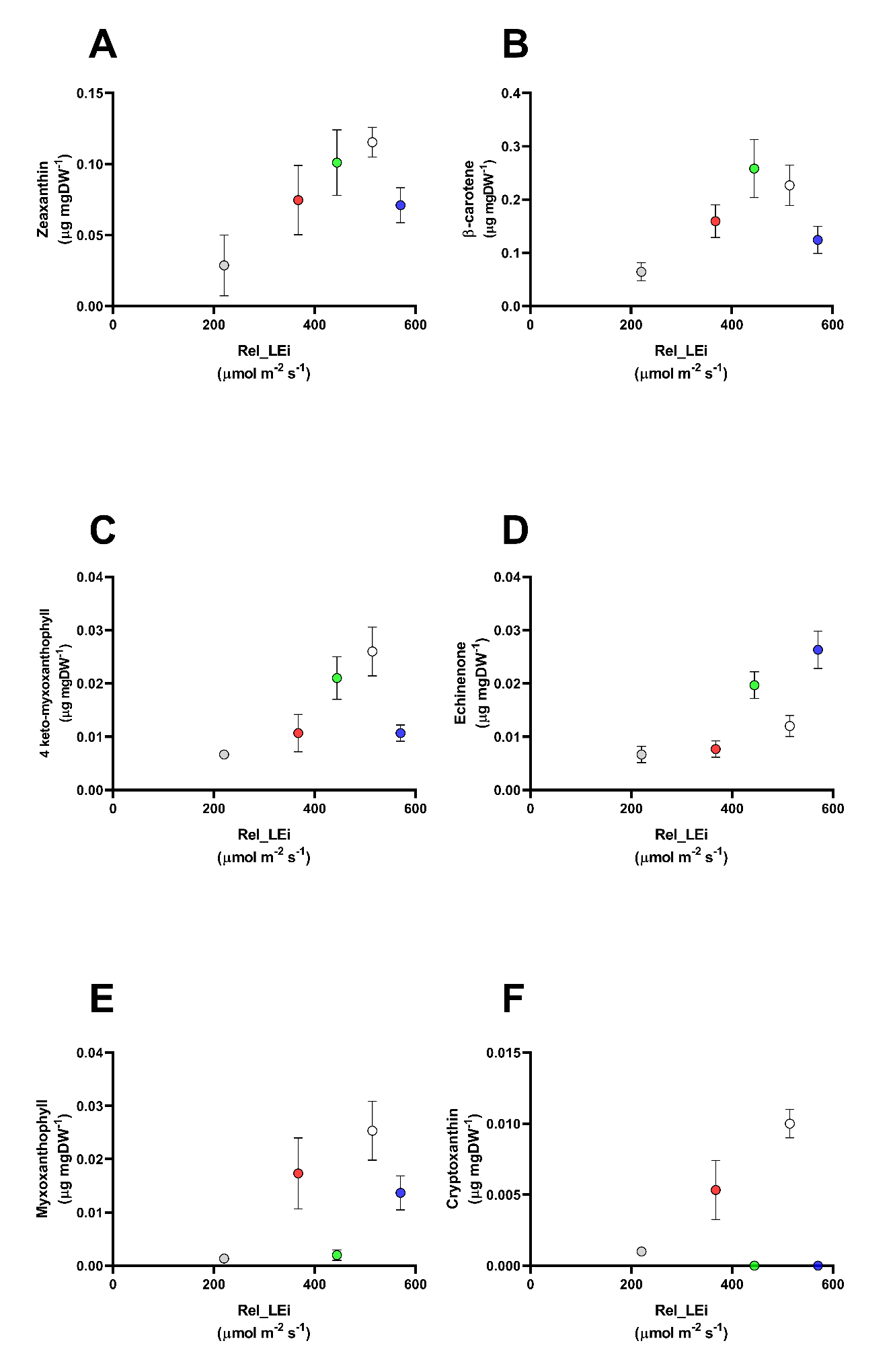


**Figure S2.**

Supplement: Supplementary file 3 — Additional file 3: Figure S2. Carotenoid concentration in the S. subsala biomass vs rel_LEi (µmol m−2 s−1). (A) Zeaxanthin (μg mg DW−1); (B) β-carotene (μg mg DW−1); (C) 4 keto-myxoxanthophyll (μg mg DW−1); (D) echinenone (μg mg DW−1); (E) myxoxanthophyll (μg mg DW−1); (F) cryptoxanthin (μg mg DW−1). Blue (BHL condition); Red (RHL condition); Green (GHL condition); White (WHL condition); grey (WLL condition). See Table 2 for rel_LEi information and calculation. [file 13068_2023_2277_MOESM3_ESM.docx]

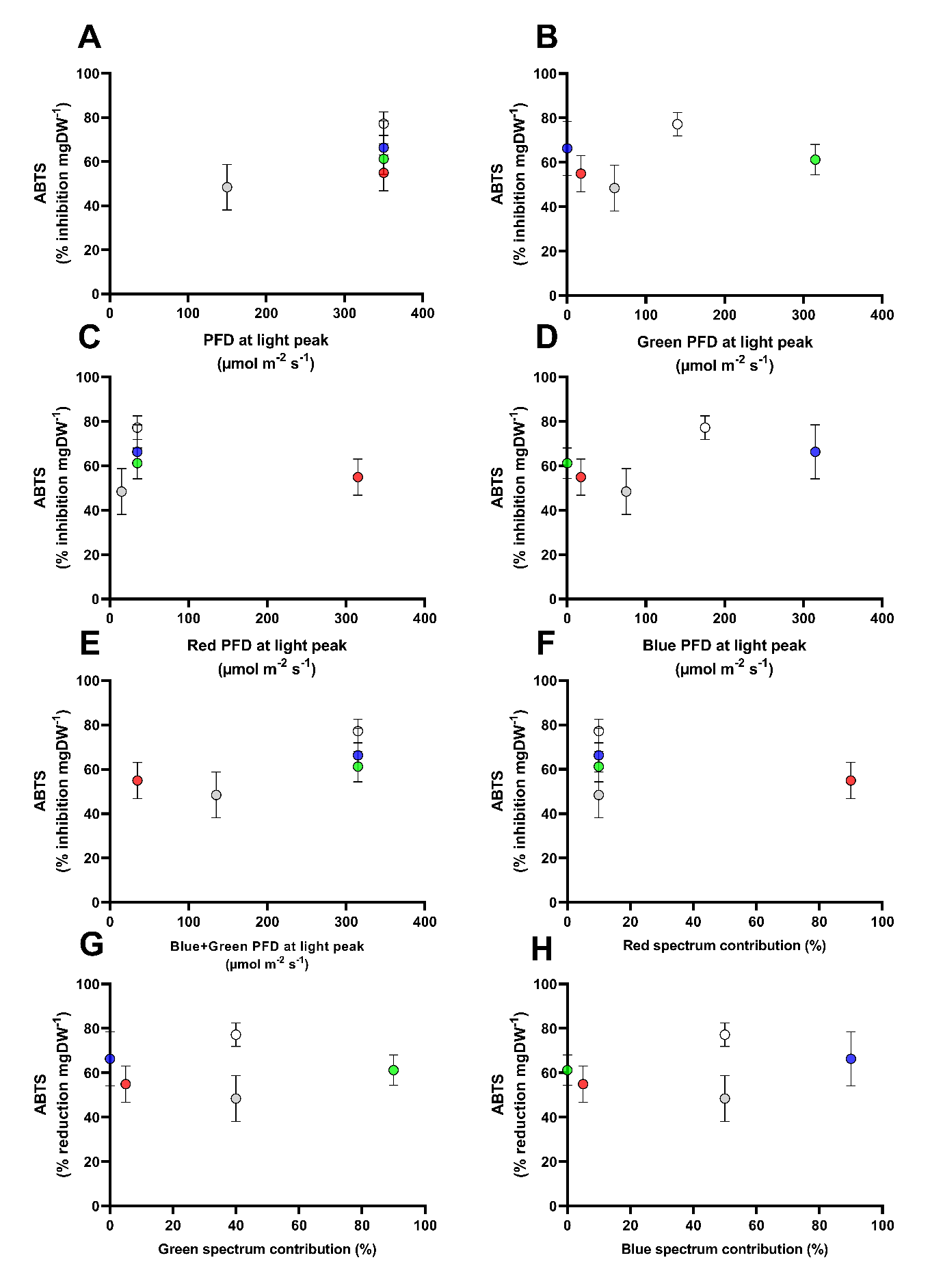


**Figure S3.**

Supplement: Supplementary file 4 — Additional file 4: Figure S3. Comparative distribution of the antioxidant property of the S. subsala biomass estimated with ABTS assay (% inhibition mgDW−1) vs different light indexes characterizing the five experimental light climates (see Table 2 and Fig. 6A): (A) ABTS vs PFD at light peak (μmol m−2 s−1); (B) ABTS vs green spectrum PFD at light peak (μmol m−2 s−1); (C) ABTS vs red spectrum PFD at light peak (μmol m−2 s−1); (D) ABTS vs blue spectrum PFD at light peak (μmol m−2 s−1); (E) ABTS vs blue + green spectra PFD at light peak (μmol m−2 s−1); (F) ABTS vs red spectrum contribution (%); (G) ABTS vs green spectrum contribution (%); (H) ABTS vs blue spectrum contribution (%). Blue (BHL condition); Red (RHL condition); Green (GHL condition); White (WHL condition); grey (WLL condition). See Table 2 for rel_LEi information and calculation. [file 13068_2023_2277_MOESM4_ESM.docx]
