# Supplementary material for: Biotechnological response curve of the cyanobacterium Spirulina subsalsa to light energy gradient
Source: Biotechnol Biofuels Bioprod. 2023 Feb 19;16:28. doi: 10.1186/s13068-023-02277-4 (PMC9940373; doi:10.1186/s13068-023-02277-4)
Supplement: Supplementary file 5 — Additional file 5: Table S2. List of the twelve antibodies used for vitamin determination applying competitive ELISA assay. [file 13068_2023_2277_MOESM5_ESM.docx]

**Table S2.** List of antibodies used for vitamins’ determination applying competitive ELISA assay.

| **Brand** | **Code** | **Target** | **Host** | **Clonality** |
| --- | --- | --- | --- | --- |
| Creative Diagnostics | CABT-B8962 | Anti-Vitamin A | Rabbit | Polyclonal |
| Cloud Clone | PAD053Ge01 | Anti-Vitamin B_1_ | Rabbit | Polyclonal |
| Cloud Clone | PAD054Ge01 | Anti-Vitamin B_2_ | Rabbit | Polyclonal |
| Cloud Clone | PAA916Ge01 | Anti-Vitamin B_6_ | Rabbit | Polyclonal |
| Fitzgerald | 20-FR19 | Anti-Vitamin B_9_ | Rabbit | Polyclonal |
| Creative Diagnostics | DPATB-H83238 | Anti-Vitamin B_12_ | Rabbit | Polyclonal |
| Cloud Clone | PAA913Ge01 | Anti-Vitamin C | Rabbit | Polyclonal |
| Cloud Clone | PAA921Ge01 | Anti-Vitamin D_2_ | Rabbit | Polyclonal |
| Cloud Clone | PAA920Ge01 | Anti-Vitamin D_3_ | Rabbit | Polyclonal |
| Creative Diagnostics | DPAB-DC3974 | Anti-Vitamin E | Rabbit | Polyclonal |
| Creative Diagnostics | DPAB-DC4159 | Anti-Vitamin H | Rabbit | Polyclonal |
| Cloud Clone | PAA926Ge01 | Anti-Vitamin K_1_ | Rabbit | Polyclonal |
